# The Combined Efficacy of a Two-Year Period of Cybernic Treatment With a Wearable Cyborg Hybrid-Assistive Limb and Leuprorelin Therapy in a Patient With Spinal and Bulbar Muscular Atrophy: A Case Report

**DOI:** 10.3389/fneur.2022.905613

**Published:** 2022-06-24

**Authors:** Hideaki Nakatsuji, Tetsuhiko Ikeda, Atsushi Hashizume, Masahisa Katsuno, Gen Sobue, Takashi Nakajima

**Affiliations:** ^1^Department of Clinical Research, National Hospital Organization Niigata National Hospital, Kashiwazaki, Japan; ^2^Department of Neurology, Nagoya University Graduate School of Medicine, Nagoya, Japan; ^3^Department of Neurology, National Hospital Organization Niigata National Hospital, Kashiwazaki, Japan; ^4^Brain and Mind Research Center, Nagoya University, Nagoya, Japan; ^5^Aichi Medical University, Nagakute, Japan

**Keywords:** spinal and bulbar muscular atrophy, hybrid assistive limb, cybernic treatment, leuprorelin acetate, anti-androgen therapy, creatine kinase, case report

## Abstract

Spinal and bulbar muscular atrophy (SBMA), also known as Kennedy's disease, is a rare, slowly progressive, incurable, and hereditary neurodegenerative disease caused by the testosterone-dependent accumulation of pathogenic polyglutamine-expanded androgen receptor protein. After extensive review, two treatments for SBMA have recently been approved in Japan; this decision was based on the results of randomized controlled trials: First, anti-androgen therapy using leuprorelin acetate (leuprorelin), a disease-modifying drug that can inhibit the progression of dysphagia but has not yet been proved to improve gait function; second, cybernic treatment with a wearable cyborg hybrid assistive limb (HAL®) (Cyberdyne Inc. Tsukuba, Japan). The HAL is an innovative walking exercise system that has been shown to significantly improve gait function in eight neuromuscular diseases without reduction in muscle function, including SBMA. It is possible that the combination of these two approaches might yield better outcomes. However, the long-term effects of such a combined approach have yet to be clinically evaluated. Here, we describe the case of a 39-year-old male with SBMA who commenced anti-androgen therapy with leuprorelin 1 year previously; this was followed by cybernic treatment with HAL. The duration of walking exercise with HAL was 20–30 min a day in one session. Over 2 weeks, the patient underwent nine sessions (one course). The efficacy of HAL was evaluated by gait function tests before and after one course of cybernic treatment. Then, leuprorelin treatment was combined with cybernic sessions every 2 months for 2 years (13 courses in total). Walking ability, as evaluated by the 2-min walk test, improved by 20.3% in the first course and peaked 10 months after the commencement of combined therapy (a 59.0% improvement). Walking function was maintained throughout the period. Generally, SBMA is characterized by moderately increased serum levels of creatine kinase (CK), reflecting neuromuscular damage; interestingly, the patient's CK levels decreased dramatically with combined therapy, indicating remarkable functional improvement. Long-term combined therapy improved the patient's gait function with a steady reduction in CK levels. The combination of leuprorelin with cybernic treatment can, therefore, improve and maintain gait function without damaging the motor unit and may also suppress disease progression.

## Introduction

Spinal and bulbar muscular atrophy (SBMA) was first described by Kennedy et al. in 11 patients from two families as a progressive proximal spinal and bulbar muscular atrophy of the late onset and described this as a sex-linked recessive trait; this condition is now known as Kennedy's disease (1). SBMA is a rare neuromuscular lower motor neuron disease with estimated prevalence of 1–2 per 100,000 males globally (2, 3). SBMA is associated with some of the features of primary myopathy and is characterized by high serum levels of creatine kinase (CK) prior to the onset of symptoms (4, 5).

Over time, SBMA gradually impairs gait function, thus leading to the need for patients to use a wheelchair ~20 years after the onset (6). Bulbar palsy symptoms occur in almost all patients, and the most common cause of death is aspiration pneumonia caused by dysphagia. Considering these symptoms, the treatment strategy for SBMA is to prevent aspiration and maintain ambulatory function. Furthermore, this disease is characterized by a slow progressive deterioration of various motor functions, thus resulting in a prolonged and gradually increasing burden on the patient. For this reason, there is a clear need to establish treatments that can alleviate each symptom and potentially cure the disease completely.

SBMA is caused by the abnormal expansion of CAG repeats in the androgen receptor (*AR*) gene on the X chromosome (7). In addition, previous neuropathological examination of 11 autopsied cases showed that the loss of lower motor neurons is associated with the nuclear accumulation of polyglutamine-expanded AR protein (8). These findings led to the development of a therapeutic strategy to prevent the nuclear accumulation of abnormal AR proteins. Further research, involving a mouse model of SBMA, showed that castration improved phenotypic expression by reducing the levels of testosterone to undetectable levels. These data indicated that the pathogenesis of this disease depends on the levels of testosterone, a known ligand for AR (9). Therefore, it was hoped that the anti-androgen therapy with leuprorelin acetate (leuprorelin), which has been used previously in the treatment of prostate and breast cancer, could be used as drug repositioning therapy without the need for castration in humans (10).

Randomized controlled clinical trials of leuprorelin for the treatment of SBMA ultimately demonstrated the benefit of this drug for the preservation of swallowing function in a patient treatment group (11, 12). In addition, some patients receiving leuprorelin exhibited a significant reduction in the accumulation of mutant ARs in scrotal skin biopsies (11).

These findings showed that leuprorelin was a potential disease-modifying drug, and, in 2017, leuprorelin was approved in Japan as an orphan drug to improve the swallowing dysfunction of patients with SBMA (13). However, the lack of improvement in limb muscle weakness and gait function in patients with SBMA remained problematic (14). Further research showed that, although leuprorelin improved dysphagia when compared to a control group, it did not improve gait function (12, 15). Consequently, leuprorelin was not considered to be a perfect disease-modifying drug (14). There was also concern that even if treatment led to an improvement in dysphagia, if muscle weakness and walking function did not improve, the patient would fall more frequently; this would lead to bone fractures and reduce the patient's quality of life and life expectancy.

There are cases of patients with SBMA being treated with various exercise therapies, ranging from high to low intensity. However, there have been no reports of improvement in terms of gait function in these cases (16–18). Furthermore, findings suggested that, when patients with SBMA are treated with conventional exercise therapy, the remaining motor neurons may activate more frequently, thus resulting in overexcitement in the motor unit and a likelihood of neuronal loss.

**Figure 1:**
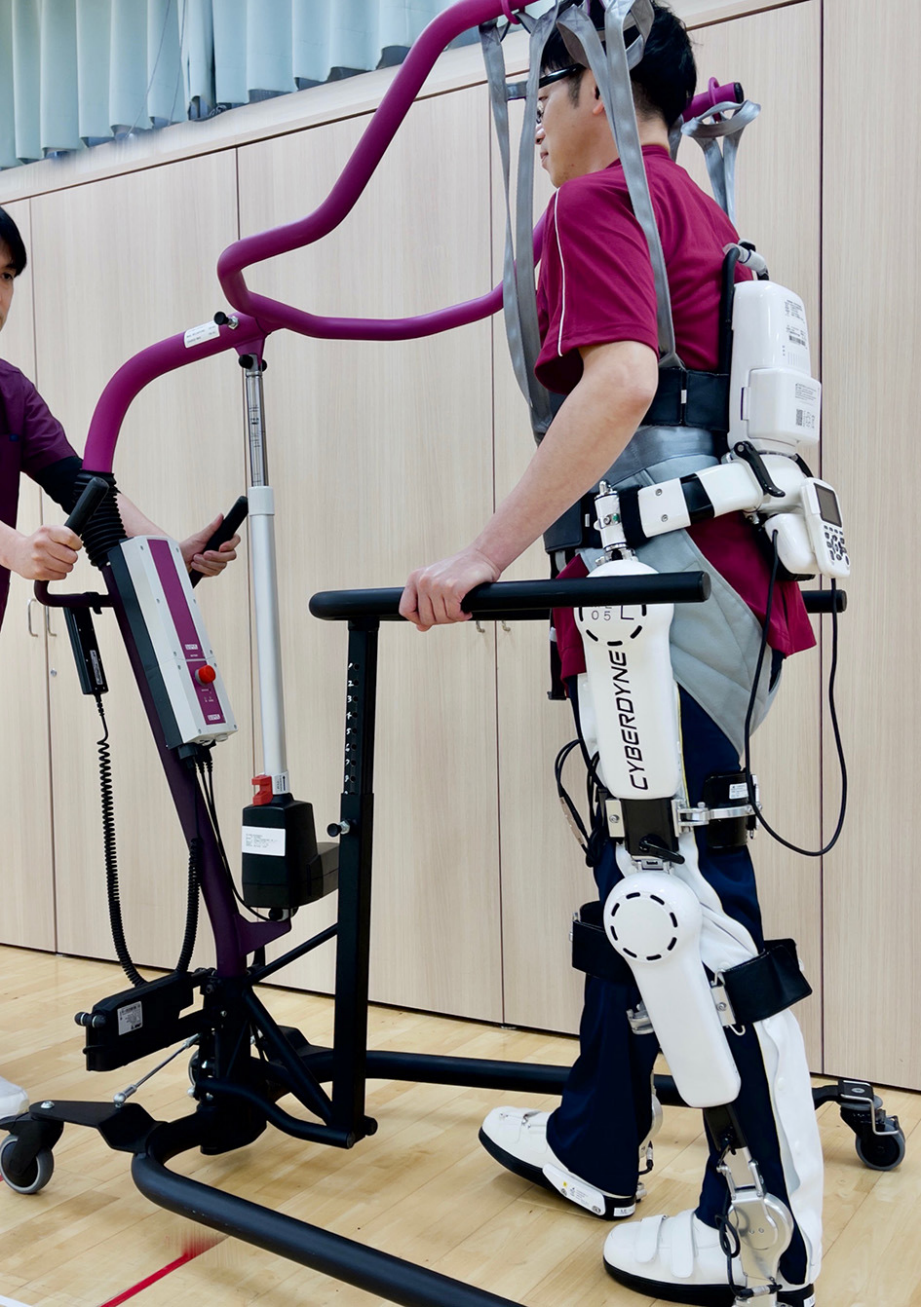
A photograph showing a hybrid-assisted limb (HAL) for medical use (lower limb type) (20). For safety reasons, we also used a mobile hoist (All-in-One®, Ropox A/S, Denmark) to prevent falls during cybernic treatment with HAL.

Sankai et al. considered the potential medical application of cybernics, an innovative technology that connects humans and devices electrically and mechanically to enhance their movements (19). Based on cybernic technology, Sankai and Nakajima developed a completely new exercise therapy that incorporated a wearable cyborg hybrid-assistive limb (HAL) for neurorehabilitation, featuring “interactive biofeedback” (iBF), which they named cybernic treatment (20) (Figure 1). The HAL and the wearer's movements are controlled by three hybrid mechanisms (20): cybernic voluntary control (CVC), cybernic autonomous control (CAC), and cybernic impedance control (CIC). The HAL controls the torque of the electric power unit based on the wearer's motor unit potentials (CVC). The wearer can walk without overloading the lower limbs. Simultaneously, the HAL and the wearer can move the lower limbs with HAL's built-in ideal gait pattern based on force plate signals and joint angle measurements (CAC). *Via* proprioception, the wearer senses that the gait patterns close to the ideal have been achieved using CIC. These mechanisms allow the wearer to repeat the successful gait in a safe manner without fatigue, thus promoting motor learning and the regeneration of walking function.

Prior to the invention of HAL, it had been thought that exercise therapy might cause the overexcitement of motor units and exacerbate diseases, such as SBMA, spinal muscular atrophy (SMA), amyotrophic lateral sclerosis (ALS), and Duchenne muscular dystrophy (DMD).

However, a randomized controlled clinical trial (NCY-3001) demonstrated that innovative HAL-based gait exercise, or cybernic treatment, was safe and efficacious when used to treat eight neuromuscular diseases, including SBMA (20). As a result, cybernic treatment was officially approved in Japan and the USA in 2015 and 2020, respectively.

As with the limitations of leuprorelin therapy when used alone (12, 15), we considered that, without the combined effect of a disease-modifying drug, such as leuprorelin, the effect of HAL alone would not last long enough to inhibit disease progression (20). Therefore, we hypothesized that the combination of leuprorelin and HAL might overcome the limitations of each of the two therapies alone, thus resulting in a synergistic effect.

Therefore, we focused on the lack of efficacy for leuprorelin when used alone to treat gait disturbance in SBMA. We hypothesized that the combination of leuprorelin and HAL would further enhance the efficacy of disease-modifying drugs, including leuprorelin. At present, Japan is the only country in which both therapies are officially approved. Clinicians are, therefore, in a unique position in that they can use leuprorelin and HAL simultaneously to treat patients with SBMA. Since conducting a randomized clinical trial of such a combined treatment is not easy, we believe that a well-designed observational study like ours is essential and have reported our data here as a case report.

## Case Description

A 39-year-old man with SBMA noticed an inability to run 12 years previously. A few years after this initial observation, the patient exhibited limb weakness with elevated levels of creatine kinase (CK), a nasal voice, tongue atrophy, hand tremor, and muscle fasciculation. None of the patient's blood relatives exhibited symptoms that were suggestive of SBMA. The patient received genetic counseling and psychological support. Genetic testing of the *AR* gene revealed 60 CAG repeats (the normal number of repeats is 9–34). Thus, the patient was diagnosed with SBMA at 29 years of age. At 38 years of age, the patient needed a railing to climb the stairs. Until then, he had never received any medical or motor exercise therapy. At this time, anti-androgen therapy was initiated; every 12 weeks, the patient was subcutaneously administered with leuprorelin acetate (11.25 mg) using a Leuplin SR Injection Kit (Takeda Pharmaceutical Company Ltd. Japan) (13). The patient's serum testosterone levels were quickly suppressed and maintained below the lower limit of the normal range. Half a year later, the patient noticed progressive limb weakness, indicating the anti-anabolic effect of the anti-androgen therapy. During this time, the patient received no form of physical therapy. One year after the patient's first injection of leuprorelin, the patient started intensive and innovative motor learning in the form of cybernic treatment with HAL; this treatment took place in a hospital with specialized rehabilitation facilities for neuromuscular diseases. At this time, the following neurological findings were noted. There was mild muscle weakness in the oris muscles and slight atrophy of the tongue. Dysarthria only involved a slight nasal voice; there was no dysphagia. The patient had a slight tremor in both upper limbs. There was mild weakness in the proximal muscles of the extremities, and the tendon reflexes of the extremities were slightly attenuated. There were no pathological reflexes, cerebellar symptoms, sensory disturbances, or autonomic symptoms. There was no joint deformity or limitations in the range of motion. At this time, his Barthel index was 100, indicating complete independence, although he used a cane to walk long distances.

**Figure 2:**
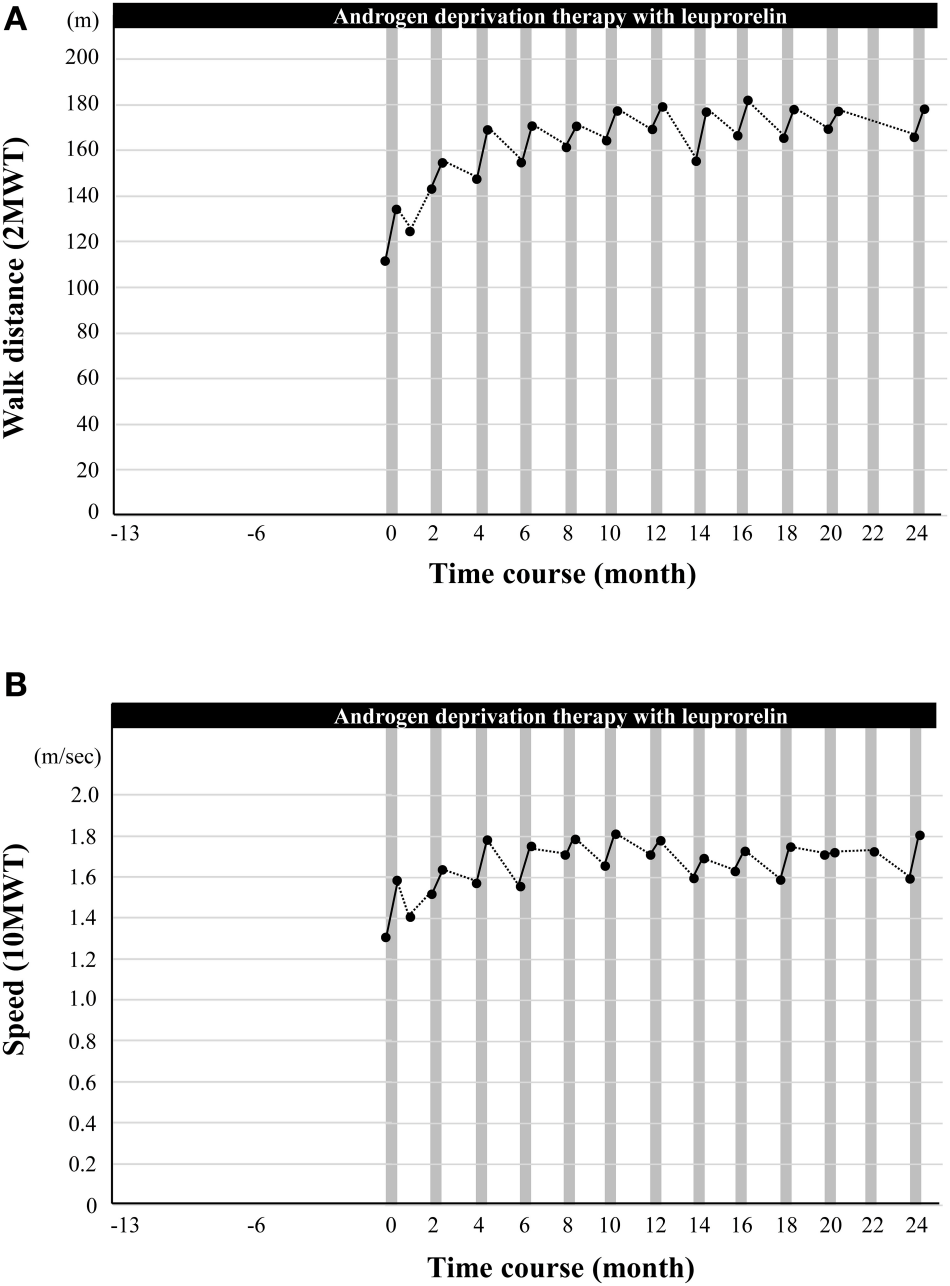
The effect of cybernic treatment with a hybrid-assisted limb (HAL) on gait function. **(A)** The black dots indicate the measured walk distance (a 2-min walk test, 2MWT). **(B)** The black dots indicate the speed (a 10-min walk test, 10MWT). Vertical gray bars indicate one course of cybernic treatment with HAL.

Walking exercise therapy with the HAL (Medical Use Lower Limb Type; Cyberdyne Inc., Tsukuba, Japan) (21) commenced after fitting the device and adjusting the torque and signal sensitivity of the motor unit potentials (Figure 1). The duration of the walking exercise with HAL was 20–30 min per day in one session. The patient completed nine HAL sessions (one course) in ~2 weeks with regular physiotherapy. Gait function was assessed by two standardized gait function tests: the 2-min walk test (2MWT), which indicates walk endurance, and the 10-min walk test (10MWT), which indicates walk speed (20). As shown in Figure 2, the patient's walking ability improved with clinical significance after the first course of cybernic treatment; the 2MWT improved from 111.5 m at the baseline to 134.2 m (+20.3%) after the first course (Figure 2A). His walking speed also improved from 1.31 to 1.58 m/s (+21.2%) (Figure 2B). These improvements are comparable to those published previously in the NCY-3001 clinical trial (20). Initially, his walking pattern was characterized by unnatural swaying while lifting his waist and legs. After the treatment, he felt the driving force of walking from behind and the swaying of his body axis decreased; furthermore, his walking became more natural. This initial improvement was highly evident. However, despite self-training at home, both the 2MWT and the 10MWT worsened mildly after 1 month (Figure 2). Fortunately, even with this deterioration, these data were better than before the onset of HAL treatment.

For the first 10 months, the gait tests prior to cybernic treatment were moderately lower than immediately after the last course of cybernic treatment. Following each course of cybernic treatment, the gait tests improved steadily to a level that was higher than immediately after the previous treatment. Ten months after the initiation of HAL, the 2MWT reached a peak at 177.3 m (+59.0%) (Figure 2A; Supplementary Video 1). From 18 months to 2 years, the 2MWT was maintained between 165 m (+46.7%) and 178 m (+59.6%). Regular cybernic treatment with HAL, combined with leuprorelin, was continued every 2 months for 2 years (13 courses in total), with the patient's willingness and satisfaction. The peak 10MWT was observed slightly earlier than in the 2MWT. The rate of improvement in the 2MWT over 2 years was higher than that in the 10MWT (+59.6% vs. +38.2%) (Figures 2A,B). Therefore, this implied that the walking endurance continued to improve with long-term cybernic treatment. Consequently, regular cybernic treatment with HAL appears to improve and maintain walking function.

**Figure 3:**
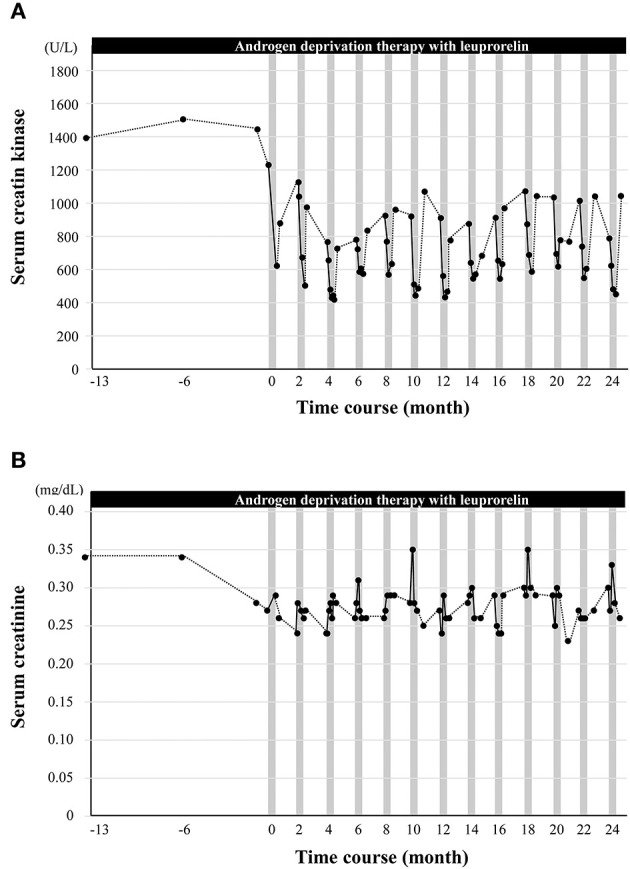
The effect of cybernic treatment with a hybrid-assisted limb (HAL) on the serum levels of creatine kinase and creatinine. **(A)** The black dots indicate the serum levels of creatine kinase. **(B)** The black dots indicate the serum levels of creatinine. Vertical gray bars indicate one course of cybernic treatment with HAL.

Serum levels of CK reflect muscle damage and are elevated in neuromuscular diseases, including SBMA (22). The patient's serum CK levels decreased from 1,230 to 622 U/L after his first course of cybernic treatment with HAL (Figure 3A). Then, the levels rose back to 878 U/L; this was still rather low at approximately two-thirds of the original baseline. Moreover, with continued cybernic treatments, the levels of CK gradually declined, corresponding to an improvement in the 2MWT. This trend was consistent in every course. Using a pedometer, the patient's total physical activity was also measured during his hospital stay; this recorded ~3,800 steps and was similar to the patient's ordinary level of daily activity. Our clinical team quickly rejected the idea that rest and immobility had reduced the patient's CK levels, which may be the case during hospitalization for acute illness. Instead, we concluded that the combination of leuprorelin therapy and HAL reduced the CK levels due to HAL's therapeutic effect and efficacy in improving gait. Conversely, the patient's serum creatinine level, a biomarker of muscle mass in SBMA (23), was maintained (Figure 3B). The improvement and maintenance of gait function, the repeated drastic reduction in serum CK levels, and the increase and maintenance of serum creatinine levels were observed only after the combined therapy of HAL and leuprorelin, and not during the period of leuprorelin monotherapy (from −13 to 0 months).

## Discussion

This report describes the first successful case in the history of SBMA treatment in which the improvement and maintenance of ambulatory motor function were observed over 2 years. Furthermore, we also observed a reduction in serum CK levels as a biomarker of muscle damage, and the maintenance of serum creatinine levels as a biomarker of muscle volume retention. Our interpretation is that these outcomes are the result of the combination of leuprorelin and HAL.

Thus far, none of the existing pharmaceutical agents, including leuprorelin, have achieved functionally efficacy with regard to muscle weakness or gait in patients with SBMA (12, 24). Consequently, it has remained unclear as to whether the levels of serum CK and creatinine, as key biomarkers, correspond to a response to treatment (5). Leuprorelin alone has been previously reported to reduce serum CK levels by ~20% after 48 weeks of treatment (24). In the present case, the reduction in CK levels after 1 year of leuprorelin administration alone was not comparable. However, we found that the levels of CK decreased from 1,230 to 622 IU/L (−51%) when combined with HAL treatment. After returning to daily life, the levels of CK only increased by a small extent. Serum creatinine data did not indicate a reduction in muscle mass; furthermore, pedometer data did not show a reduction in muscle damage due to rest. Moreover, a decreasing trend of CK was observed after each course of HAL. In other words, we can assume that the levels of CK were reduced by the combined therapeutic effects of leuprorelin and HAL. The NCY-3001 study has previously tested the effects and safety of cybernic treatment for progressive neuromuscular diseases, including SBMA but did not confirm muscle damage directly by measuring serum CK levels. In the present case report, the safety of HAL treatment combined with leuprorelin was further reinforced by the reduction in serum CK levels. However, in the patient described herein, we found that the combination of leuprorelin and HAL reduced serum CK levels and maintained the levels of creatinine; these effects correlated with an improvement in gait function. Therefore, the results of this case study suggest that these simultaneous changes in serum CK and creatinine are valid therapeutic indicators for SBMA (5).

Our results indicate a clear therapeutic effect on the pathological mechanism of SBMA, i.e., the suppression of motor neurons and muscle fiber injury were correlated with an improvement in function. We believe that this fact may be explained by the therapeutic mechanism, especially the iBF. The iBF selects the neural network that inhibits higher central controls to the pathological motor units and increases commands to the healthier motor units (20, 25, 26). Cybernic treatment might improve motor function and inhibit the degeneration of motor neurons.

Furthermore, this combined treatment led to an improvement of ambulatory function that was maintained for 2 years, with high levels of patient satisfaction. During a previous study of the natural history of SBMA, the 6-min walk test (6MWT) was found to decrease by 11.3% per year (27). The 2MWT is a simplified version of the 6MWT that correlates accurately with a lower patient burden (28); the rate of decrease is expected to be similar. We found that the 2MWT at the start of HAL was 111.5 m. If we apply a rate of decrease based on the natural history of this disease, we can estimate that the 2MWT would have decreased to ~88 m after 2 years. However, the actual value increased to 178.0 m; this was +102% of the estimated value and, therefore, represents a remarkable treatment effect.

Leuprorelin was approved in Japan for the treatment of SBMA (13) primarily because of its biological mechanism as a disease-modifying agent that acts on the primary etiology of SBMA and, secondly, because of its proven clinical efficacy for dysphagia in clinical trials (12, 24). Clinical trials of leuprorelin monotherapy did not demonstrate any improvement in ambulatory function.

In addition to SMBA, similar problems were observed in the lack of improvement in gait function, following the administration of exon-skipping therapy after the onset of DMD (29) and the lack of effect of SMN protein-enhancing treatment in patients with SMA several years after the onset (30). Therefore, to significantly amplify the efficacies of disease-modifying drugs acting on the primary etiologies of DMD and SMA, it is essential that we combine drug therapy and cybernic treatment with HAL.

This observational single case report clarifies the clinical efficacy of a combination of leuprorelin treatment for dysphagia and HAL treatment for gait disturbance. However, there is a limitation to be considered in that this combination of therapies was not compared statistically to monotherapies, HAL or leuprorelin. Therefore, it is still unclear whether the combined treatment of leuprorelin and HAL can constantly reduce serum CK levels and maintain ambulatory function over the long term. Consequently, it appears to be crucial that we conduct future RCTs to compare the combined treatment to HAL monotherapy with regard to changes in 2MWT, serum CK, and creatinine as outcome measures. However, this type of randomized trial may be challenging in a region where both treatments are officially approved. A comparative study may be possible in observational cohorts with more patients in Japan.

## Conclusion

In this case report, the novel form of combined therapy overcame the apparent limitations of disease-modifying drugs acting on primary etiology and improved the gait function of patients with SBMA over the long term. Although this is only a single case report, the results are groundbreaking. If this phenomenon can be generalized, it may be possible to improve the treatment efficacy of DMD and SMA, as well as SBMA, by introducing combined therapy with HAL.

## Data Availability Statement

The original contributions presented in the study are included in the article/Supplementary Material, further inquiries can be directed to the corresponding author.

## Ethics Statement

The studies involving human participants were reviewed and approved by the Ethics Committee of National Hospital Organization Niigata National Hospital. The patients/participants provided their written informed consent to participate in this study. Written informed consent was obtained from the individual(s) for the publication of any potentially identifiable images or data included in this article.

## Author Contributions

HN drafted and revised the manuscript and was responsible for the acquisition of data, the analysis of data, the interpretation of data, research project execution, study design, and concept. TI and AH acquired the data. MK revised the manuscript and analyzed/interpreted the data. GS was responsible for analysis/interpretation of the data. TN revised the manuscript, acquired/analyzed/interpreted the data, executed the research project, and was also involved with the study design and concept. All authors contributed to the article and approved the submitted version.

## Funding

This research was supported by Grants-in Aid from the Research Committee of CNS Degenerative Diseases (Reference: 20FC1049), Research on Dissemination of Best Practicable Care for Muscle Dystrophy (Reference: 21FC1006), and Research on Policy Planning and Evaluation for Rare and Intractable Diseases, Health, Labour and Welfare Sciences Research Grants, the Ministry of Health, Labour and Welfare, Japan.

## Conflict of Interest

AH and GS received royalties from Takeda Pharmaceutical Co., Ltd. MK received a speaker's fee, a research grant, and royalties from Takeda Pharmaceutical Co., Ltd. The remaining authors declare that the research was conducted in the absence of any commercial or financial relationships that could be construed as a potential conflict of interest.

## Publisher's Note

All claims expressed in this article are solely those of the authors and do not necessarily represent those of their affiliated organizations, or those of the publisher, the editors and the reviewers. Any product that may be evaluated in this article, or claim that may be made by its manufacturer, is not guaranteed or endorsed by the publisher.

## Supplementary Material

Supplementary Video 1 Changes in gait speed and endurance in the 2MWT after commencing combined therapy. This video clip compares the baseline (an upper row) with a time point 10 months after the commencement of treatment (a lower row). The first 20 s (a left column) and the last 20 s (a right column) of the test can be observed. The lower left video (10 months later and the first 20 s in the 2MWT) shows dramatic gait improvement in speed compared to the baseline (the upper left video). The lower right video shows a dramatic improvement in gait endurance compared to the baseline (the upper right video). The patient used a mobile hoist (All-in-One®, Ropox A/S, Denmark) to prevent falls during the 2MWT. In this video, the same person in the team of evaluators pulls the mobile hoist according to the patient's gait but is careful to never strain the patient's sling and never affect the patient's gait.

## References

[1] Kennedy WR Alter M Sung JH. Progressive proximal spinal and bulbar muscular atrophy of late onset. A sex-linked recessive trait. Neurology. (1968) 18:671–80. 10.1212/WNL.18.7.671 4233749

[2] Guidetti D Sabadini R Ferlini A Torrente I. Epidemiological survey of X-linked bulbar and spinal muscular atrophy, or Kennedy disease, in the province of Reggio Emilia, Italy. Eur J Epidemiol. (2001) 17:587–91. 10.1023/A:1014580219761 11949733

[3] Finsterer J. Perspectives of Kennedy's disease. J Neurol Sci. (2010) 298:1–10. 10.1016/j.jns.2010.08.025 20846673

[4] Sorenson EJ Klein CJ. Elevated creatine kinase and transaminases in asymptomatic SBMA. Amyotroph Lateral Scler. (2007) 8:62–4. 10.1080/17482960600765040 17364438

[5] Querin G Bede P Marchand-Pauvert V Pradat PF. Biomarkers of spinal and bulbar muscle atrophy (SBMA): acomprehensive review. Front Neurol. (2018) 9:844. 10.3389/fneur.2018.00844 30364135 PMC6191472

[6] Atsuta N Watanabe H Ito M Banno H Suzuki K Katsuno M. Natural history of spinal and bulbar muscular atrophy (SBMA): a study of 223 Japanese patients. Brain. (2006) 129:1446–55. 10.1093/brain/awl096 16621916

[7] La Spada AR Wilson EM Lubahn DB Harding AE Fischbeck KH. Androgen receptor gene mutations in X-linked spinal and bulbar muscular atrophy. Nature. (1991) 352:77–9. 10.1038/352077a0 2062380

[8] Adachi H Katsuno M Minamiyama M Waza M Sang C Nakagomi Y. Widespread nuclear and cytoplasmic accumulation of mutant androgen receptor in SBMA patients. Brain. (2005) 128:659–70. 10.1093/brain/awh381 15659427

[9] Katsuno M Adachi H Kume A Li M Nakagomi Y Niwa H. Testosterone reduction prevents phenotypic expression in a transgenic mouse model of spinal and bulbar muscular atrophy. Neuron. (2002) 35:843–54. 10.1016/S0896-6273(02)00834-6 12372280

[10] Katsuno M Adachi H Doyu M Minamiyama M Sang C Kobayashi Y. Leuprorelin rescues polyglutamine-dependent phenotypes in a transgenic mouse model of spinal and bulbar muscular atrophy. Nat Med. (2003) 9:768–73. 10.1038/nm878 12754502

[11] Banno H Katsuno M Suzuki K Takeuchi Y Kawashima M Suga N. Phase 2 trial of leuprorelin in patients with spinal and bulbar muscular atrophy. Ann Neurol. (2009) 65:140–50. 10.1002/ana.21540 19259967

[12] Hashizume A Katsuno M Suzuki K Banno H Takeuchi Y Kawashima M. Efficacy and safety of leuprorelin acetate for subjects with spinal and bulbar muscular atrophy: pooled analyses of two randomized-controlled trials. J Neurol. (2019) 266:1211–21. 10.1007/s00415-019-09251-x 30847645

[13] PMDA. Leuplin SR Injection Kit 11.25mg. (2017). Available online at: https://www.pmda.go.jp/PmdaSearch/iyakuDetail/400256_2499407G3030_1_07 (accessed February 6, 2022).

[14] Arnold FJ Merry DE. Molecular mechanisms and therapeutics for SBMA/Kennedy'sdisease. Neurotherapeutics. (2019) 16:928–47. 10.1007/s13311-019-00790-9 31686397 PMC6985201

[15] Hashizume A Katsuno M Suzuki K Hirakawa A Hijikata Y Yamada S. Long-term treatment with leuprorelin for spinal and bulbar muscular atrophy: natural history-controlled study. J Neurol Neurosurg Psychiatry. (2017) 88:1026–32. 10.1136/jnnp-2017-316015 28780536

[16] Preisler N Andersen G Thogersen F Crone C Jeppesen TD Wibrand F. Effect of aerobic training in patients with spinal and bulbar muscular atrophy (Kennedy disease). Neurol. (2009) 72:317–23. 10.1212/01.wnl.0000341274.61236.02 19171827

[17] Shrader JA Kats I Kokkinis A Zampieri C Levy E Joe GO. A randomized controlled trial of exercise in spinal and bulbar muscular atrophy. Ann Clin Transl Neurol. (2015) 2:739–47. 10.1002/acn3.208 26273686 PMC4531056

[18] Heje K Andersen G Buch A Andersen H Vissing J. High-intensity training in patients with spinal and bulbar muscular atrophy. J Neurol. (2019) 266:1693–7. 10.1007/s00415-019-09316-x 31004213

[19] Sankai Y Suzuki K Hasegawa Y. Cybernics: Fusion of Human, Machine and Information Systems. Tokyo: Springer (2014).

[20] Nakajima T Sankai Y Takata S Kobayashi Y Ando Y Nakagawa M. Cybernic treatment with wearable cyborg Hybrid Assistive Limb (HAL) improves ambulatory function in patients with slowly progressive rare neuromuscular diseases: a multicentre, randomised, controlled crossover trial for efficacy and safety (NCY-3001). Orphanet J Rare Dis. (2021) 16:304. 10.1186/s13023-021-01928-9 34233722 PMC8261928

[21] PMDA. HAL For Medical Use (Lower Limb Type). (2015). Available online at: https://www.pmda.go.jp/files/000216634.pdf (accessed February 6, 2022).

[22] Soraru G D'Ascenzo C Polo A Palmieri A Baggio L Vergani L. Spinal and bulbar muscular atrophy: skeletal muscle pathology in male patients and heterozygous females. J Neurol Sci. (2008) 264:100–5. 10.1016/j.jns.2007.08.012 17854832

[23] Hijikata Y Hashizume A Yamada S Inagaki T Ito D Hirakawa A. Biomarker-based analysis of preclinical progression in spinal and bulbar muscular atrophy. Neurol. (2018) 90:e1501–e9. 10.1212/WNL.0000000000005360 29572281

[24] Katsuno M Banno H Suzuki K Takeuchi Y Kawashima M Yabe I. Efficacy and safety of leuprorelin in patients with spinal and bulbar muscular atrophy (JASMITT study): a multicentre, randomised, double-blind, placebo-controlled trial. Lancet Neurol. (2010) 9:875–84. 10.1016/S1474-4422(10)70182-4 20691641

[25] Nakajima T. Nerve function recovery mechanism by the robot suit. Clin Neurosci. (2016) 34:936–7. Available online at: https://scholar.google.com/scholar?cluster=1089814798738094105

[26] Nakajima T. Innovative technology, clinical trials and the subjective evaluation of patients: the cyborg-type robot HAL and the treatment of functional regeneration in patients with rare incurable neuromuscular diseases in Japan. In: Brucksch S Sasaki K editors. Humans and Devices in Medical Contexts: Case Studies From Japan. Singapore: Springer Singapore (2021). p. 281–310.

[27] Takeuchi Y Katsuno M Banno H Suzuki K Kawashima M Atsuta N. Walking capacity evaluated by the 6-minute walk test in spinal and bulbar muscular atrophy. Muscle Nerve. (2008) 38:964–71. 10.1002/mus.21077 18642379

[28] Andersen LK Knak KL Witting N Vissing J. Two- and 6-minute walk tests assess walking capability equally in neuromuscular diseases. Neurology. (2016) 86:442–5. 10.1212/WNL.0000000000002332 26740680

[29] Randeree L Eslick GD. Eteplirsen for paediatric patients with Duchenne muscular dystrophy: a pooled-analysis. J Clin Neurosci. (2018) 49:1–6. 10.1016/j.jocn.2017.10.082 29254734

[30] Mercuri E Darras BT Chiriboga CA Day JW Campbell C Connolly AM. Nusinersen versus sham control in later-onset spinal muscular atrophy. N Engl J Med. (2018) 378:625–35. 10.1056/NEJMoa1710504 29443664

